# Enhanced thermoelectric performance of a chalcopyrite compound CuIn_3_Se_5−*x*_Te_*x*_ (*x* = 0~0.5) through crystal structure engineering

**DOI:** 10.1038/srep40224

**Published:** 2017-01-06

**Authors:** Yufu Lu, Shaoping Chen, Wenchang Wu, Zhengliang Du, Yimin Chao, Jiaolin Cui

**Affiliations:** 1 Materials Science and Engineering College, Taiyuan University of Technology, Taiyuan, 030024, China; 2 School of Materials & Chemical Engineering, Ningbo University of Technology, Ningbo, 315016, China; 3 School of Chemistry, University of East Anglia, Norwich NR4 7TJ, United Kingdom

## Abstract

In this work the chalcopyrite CuIn_3_Se_5−*x*_Te_*x*_ (*x* = 0~0.5) with space group 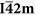 through isoelectronic substitution of Te for Se have been prepared, and the crystal structure dilation has been observed with increasing Te content. This substitution allows the anion position displacement ∆*u* = 0.25-*u* to be zero at *x* ≈ 0.15. However, the material at *x* = 0.1 (∆*u* = 0.15 × 10^−3^), which is the critical Te content, presents the best thermoelectric (TE) performance with dimensionless figure of merit ZT = 0.4 at 930 K. As *x* value increases from 0.1, the quality factor *B*, which informs about how large a ZT can be expected for any given material, decreases, and the TE performance degrades gradually due to the reduction in *n*_H_ and enhancement in *κ*_L_. Combining with the ZTs from several chalcopyrite compounds, it is believable that the best thermoelectric performance can be achieved at a certain ∆*u* value (∆*u* ≠ 0) for a specific space group if their crystal structures can be engineered.

Thermoelectric (TE) materials have their potential applications in power generation and cooling systems, therefore, they have received great attention in recent decades. The TE performance of the materials is characterized by the dimensionless figure of merit (Z*T*), Z*T* = *Tα*^2^*σ*/*κ* = *Tα*^2^*σ*/ (*κ*_L_ + *κ*_*e*_), here *T* is the absolute temperature, and *α, σ, κ, κ*_L_ and *κ*_*e*_ are the Seebeck coefficient, electrical conductivity, total thermal conductivity, lattice contribution and electronic part respectively. However, it is difficult to be improved as the *α, σ* and *κ*_e_ are usually coupled with each other strongly, which makes an independent property manipulation challenging for enhancing *ZT*.

In order to significantly improve the TE performance or ZT value, it is strongly necessary to explore effective strategies, in addition to those like nanostructure engineering^1^,^2^,^3^ and band structure engineering^4^,^5^,^6^. Under the guidance of such strategies, many new materials with high TE performance have been developed. The typical materials are those with high crystal symmetry structure like PbTe^4^,^5^,^6^, SnSe^7^,^8^, Mg_2_Si_1−*x*_Sn_*x*_ solid solutions^9^,^10^, and half-Heusler (HH) alloys^11^.

Beyond those solid solutions, non-cubic diamond-like compounds, such as CuGa(In)Te_2_, ZnSnP_2_ and Cu_2_ZnSnS_4_, have also high power factors and ZTs if band convergence can be realized^12^,^13^. The band converges only when the tetragonal deformation parameter *η (η* = *c*/2*a*, where *c* and *a* are the lattice parameters) becomes unity or the crystal field splitting Δ_CF_ approaches zero. However, deep investigations have revealed that as *η* approaches unity the chalcopyrite compounds often give low electrical conductivity and carrier concentration, due to the electrical inactivity caused by the attractive interaction between intrinsic donor-acceptor defect pairs^14^,^15^,^16^. In addition, the cation–anion bond lengths in many chalcopyrite compounds are around 0.26–0.28 nm^17^,^18^, a tiny disturbance of periodical crystal structure could have a profound impact on the phonon scattering mechanism. When *η* = 1 (Δ_CF_ = 0) non-cubic diamond-like compounds become pseudocubic ones^12^,^13^. In this case, the least lattice distortion can be obtained and phonon scattering in point defect gets the minimum. As a result, the highest lattice part *κ*_L_ can be attained^19^.

The aforementioned different findings suggest that the impact of crystal structure on TE performance seems to be much more complex than what we anticipated. The origin might be the presence of different space groups for each compound caused by the vacancy that exists in some chalcopyrites with different site assignments^20^. For example, in CuIn_3_Se_5_ the sum of cation atoms is less than that of anion atoms, therefore, the structure formula of CuIn_3_Se_5_ per unit cell is represented by Cu_1.6_In_4.8_□_1.6_Se_8_ where □ denotes structure vacancy^20^. Therefore, the candidate space groups for CuIn_3_Se_5_ can be 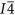, 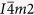, 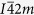, 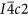 20. Since different space group gives different chemical bonding, which gives rise to different physical properties, it is strongly necessary to tune the TE performance through engineering the crystal structure.

Although CuIn_3_Se_5_ have several space groups, the previous investigations have revealed that it still is a compound with defect non-cubic chalcopyrite structure^21^,^22^,^23^,^24^,^25^,^26^ with *η* = 0.997~1.013^27^,^28^. Therefore, it is possible to engineer its crystal structure through manipulating its chemical compositions without changing its chalcopyrite structure. The tetragonal deformation parameter *η* is directly related to the anion position displacement *u* in chalcopyrites I_(*m*−3)_III_(*m*+1)_VI_2*m*_, here *m* = 4, 5, 6, 7, 8, 9 …; I = Cu, Ag; III = Al, Ga, In; VI = Te, Se, S^29^,^30^,^31^, *u* (or ∆*u* = 0.25-*u*) tends to be 0.25 (or zero) as *η* approaches unity^31^. In addition, the anion position displacement *u* in CuIn_3_Se_5_ fluctuates around 0.2435~0.2515^27^,^28^, which facilitates us to examine the ∆*u* related TE performance as ∆*u* is around zero.

In this work, the CuIn_3_Se_5_-based compounds have been prepared, and their crystal structures through isoelectronic substitution of Te with lower electronegativity (2.1) for Se (2.55) have been engineered. It is believable that such an isoelectronic substitution is very important, because it tends to elongate the bond lengths *d*_Cu-Se_ and *d*_In-Se_ due to an increased repulsion between Cu- or In-Te bonds. Therefore, it has potential to manipulate the ∆*u* value, and optimize their TE performance.

## Experimental

### Sample preparations

Four elements Cu, In, Te and Se with a purity of 99.999% were loaded into different silica tubes in vacuum according to the formula CuIn_3_Se_5−*x*_Te_*x*_ (*x* = 0, 0.05, 0.1, 0.2, 0.5), and then melted at 1273 K for 24 h, followed by a rapid cooling to RT in water. The as-solidified ingots were pulverized and then ball milled in stainless steel bowls containing benzinum at a rotation rate of 350 rpm for 5 h. The dried powders were sintered using spark plasma sintering apparatus (SPS-1030) under a pressure of 60 MPa and at the highest temperature of ~950 K. The total sintering time is about 5 min. The densities (*d*) of the sintered samples (5.43~5.48 × 10^3^ kg/m^3^), which are more than 95% theoretical values^24^, were measured using Archimedes’ method. The sintered block with the sizes of ϕ20 mm × 3.0 mm was cut into 3 mm-wide slices measuring 2.5 mm × 12 mm for electrical property measurement, and that with ϕ~10.0 mm × 2.0 mm was obtained for thermal diffusivity measurement.

### Structural analyses

The structural analysis of the powders was made by powder X-ray diffractometer (D8 Advance) operating at 50 kV and 40 mA. Cu Ka radiation (λ = 0.15406 nm) and a scan rate of 4° min^−1^ were used to record the patterns in the range from 10° to 140°.

In order to gain a deep understanding of the crystal structure, the microstructures of the samples (*x* = 0, 0.1) have been examined by using high resolution transmission electron microscopy (HRTEM), and pure CuIn_3_Se_5_ was examined for comparison. HRTEM images were obtained at 220 kV using JEM-2010F (Field emission TEM).

### TE transport property measurements

The Hall coefficient (*R*_h_) measurements at room temperature (RT) were conducted on a Physical Property Measurement System (PPMS, Model-9) using a four probe configuration with a magnetic field sweeping between ±2.0 T, and were performed on rectangular samples with size 2 × 2 × 7 mm^3^. The Hall carrier concentrations (*n*_H_) and mobility *μ* were subsequently calculated based on the following formulae *n*_H_ = −1/(*e*|*R*_h_|), *μ* = |*R*_h_|*σ*, where *e* is the electron charge. The electrodes were fine copper wires for current and Hall voltage measurements, and the contacts were made of silver paste.

The Seebeck coefficients (*α*) and electrical conductivities (*σ*) were measured as a function of temperature using ULVAC ZEM-3 instrument under helium atmosphere from RT to ~930 K. A temperature difference of approximately 5 °C was applied between the two terminals of the sample in order to measure the Seebeck coefficient, whereas the electrical conductivity was measured using the four-probe method. The measurement uncertainties are 6% for both the electrical conductivity and Seebeck coefficient. The thermal conductivities (*κ*) at RT~930 K were calculated as the product of the material densities, specific heats and thermal diffusivities (with uncertainty below 10%), which was measured using TC-1200RH. The heat capacities (*C*_*p*_) were estimated using Dulong–Petit rule, *C*_v_ = 3*nR* (where *n* is the number of atoms per formula unit, *R* is the gas constant). The total uncertainty for *ZT* is ~18%. The lattice contributions (*κ*_L_) were obtained by subtracting the electronic part (*κ*_e_) from the total *κ*, i.e., *κ*_L_ = *κ* − *κ*_e_. Here *κ*_e_ is expressed by the Wiedemann–Franz law, *κ*_e_ = *L*_0_*σT*, where *L*_0_ is the Lorenz number, estimated at 1.5 × 10^−8^ WΩK^−2^ for not fully degenerate environment of semiconductors^12^.

The parameters were finalized after several repeated measurements using different samples.

## Results and Discussion

### Structural analyses

**Figure 1:**
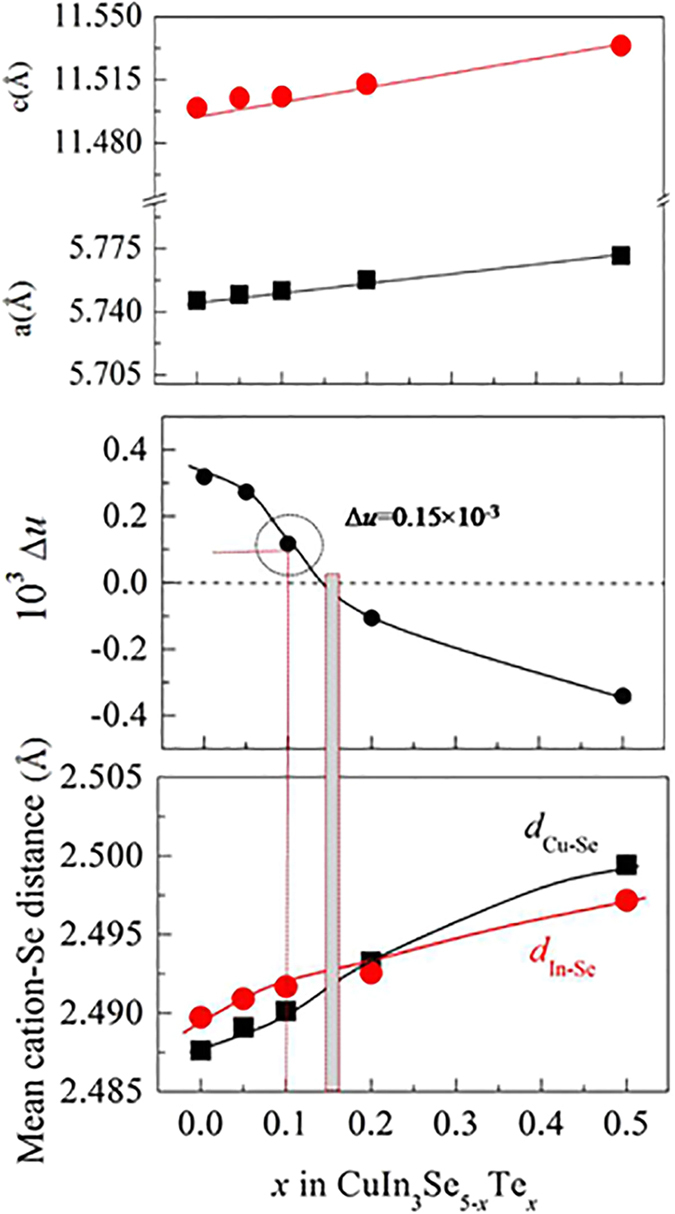
(**a**) Lattice constants *a* and *c* as a function of Te content (*x* value) in CuIn_3_Se_5−*x*_Te_*x*_, they all increase linearly with *x* value; (**b**) Anion position displacement ∆*u* = 0.25−*u*, which decreases with *x* value. At *x* = 0.1, ∆*u* = 0.15 × 10^−3^, while at *x* ≈ 0.15, ∆*u* = 0; (**c**) Mean cation-Se distance *d*_Cu-Se_ and *d*_In-Se_ as a function of Te content (*x* value), at *x* ≈ 0.15, i.e. ∆*u* = 0, the difference between *d*_Cu-Se_ and *d*_In-Se_ gets the minimum.

The x-ray diffraction patterns (Fig. S1) shows that the materials can be indexed as a tetragonal cell^20^ (PDF:51-1221) with *a* = 5.7461~5.7709 and *c* = 11.4996~11.5340 Å, without any impurity phases identified in all the composition range (*x* = 0~0.5). In XRD patterns several diffraction peaks are apparent, namely: (110), (202) or (210) and (114), which are characteristic of the CuIn_3_Se_5_ phase with the space group 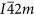 20,32,33,34,35. The lattice parameters *a* and *c*, which are in agreement with those reported^24^,^25^,^35^, both increase linearly with Te content increasing, see Fig. 1a. indicating that the chalcopyrite lattice suffers dilation upon Te substitution for Se. Since the tetragonal deformation parameter *η* and *u* are also directly related to the mean cation-anion distances *d*_I–VI_ and *d*_III–VI_ in chalcopyrite compounds^31^, an equal *d*_I–VI_ and *d*_III–VI_ values can be obtained as *η* (or *u*) approaches unity (or 0.25)^31^. The calculated ∆*u, d*_Cu-Se_ and *d*_In-Se_ values are presented in Fig. 1b,c. With the Te content increasing, the ∆*u* decreases gradually from 0.32 × 10^−3^ to −0.34 × 10^−3^, and it approaches zero at *x* ≈ 0.15 (Fig. 1b), at which *d*_Cu-Se_ roughly equals to *d*_In-Se_ (Fig. 1c).

**Figure 2:**
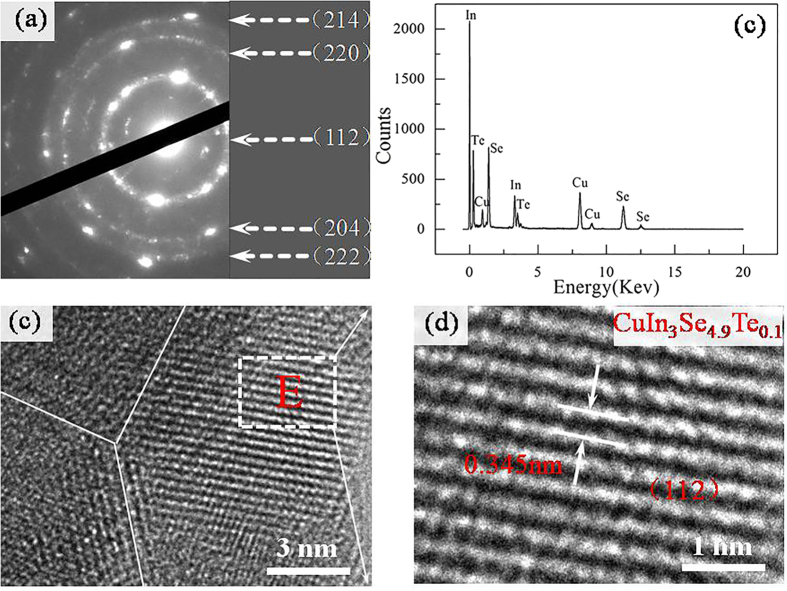
High resolution transmission electron microscopy (HRTEM) image observed in CuIn_3_Se_4.9_Te_0.1_, (**a**) The selected area electron diffraction (SAED) pattern; (**b**) Chemical compositions analyses using EDS spectra; (**c**) High resolution TEM image, showing a conventional polycrystalline structure consisting of many nano-domains; (**d**) Magnified high resolution TEM image, which shows that the spacing between (112) crystal planes is about 0.345 nm.

In order to confirm the dilation of the crystal structure upon Te substitution, the microstructure analysis using HRTEM for the sample with *x* = 0.1 is shown in Fig. 2, while that for Te-free sample is shown in Fig. S2 for comparison. Figure 2a is the selected area electron diffraction (SAED) pattern, and Fig. 2b,c are the EDS measurement results and high resolution image respectively. It is apparent that the materials show a conventional polycrystalline structure consisting of many nano-domains. The boundaries between nano-domains are not coherent, and ambiguously terminate at a grown domain 15~20 nm in size. Fig. 2d is the magnified high resolution TEM image, which shows that the *d* spacing between (112) crystal planes is about 0.345 nm. Figure S2a is SAED pattern, and Fig. S2b,c are the EDS measurement results and high resolution image for Te-free sample. Figure S2d is the magnified high resolution TEM image, which shows that the *d* spacing between (112) crystal plane is about 0.335 nm. An increase in *d* spacing upon Te substitution is resulted from the dilation of the crystal lattice, and is in accordance with the elongated lattice constants from XRD analyses.

### Transport properties and TE performance

**Figure 3:**
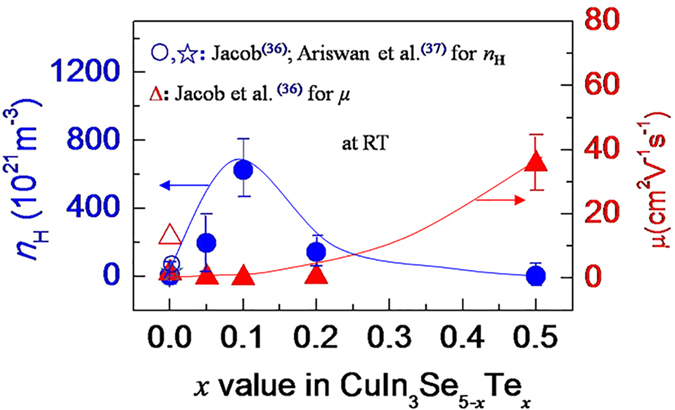
Measured Hall carrier concentration (*n*_H_) and mobility (*μ*) against *x* value in CuIn_3_Se_5−*x*_Te*_x_*. The results from Jocob^36^, Ariswan *et al*.^37^ are presented for comparison.

Although the isoelectronic substitution of Te for Se creates no extra electrons or holes, the dilation of the crystal structure upon such a substitution should have a profound impact on the structural and transport properties. In order to verify this conclusion, the Hall coefficients were measured at room temperature (RT), and the Hall carrier concentrations (*n*_H_) and mobility (*μ*) were then calculated. The results are shown in Fig. 3. The measured *n*_H_ value, which is 4.11 × 10^21^ m^−3^ at *x* = 0, comparable to those from Jacob (3.40 × 10^21^ m^−3^)^36^ and Ariswan (1.16 × 10^21^ m^−3^)^37^, reaches the highest (6.27 × 10^23^ m^−3^) at *x* = 0.1 and then reduces to 1.58 × 10^21^ m^−3^ at *x* = 0.5. While the mobility (*μ*) tends to increase from 1.62 cm^2^ V^−1^ s^−1^, which is less than that from Jacob (12.8 cm^2^ V^−1^ s^−1^)^36^, to the highest 35.48 cm^2^ V^−1^ s^−1^ at *x* = 0.5.

**Figure 4:**
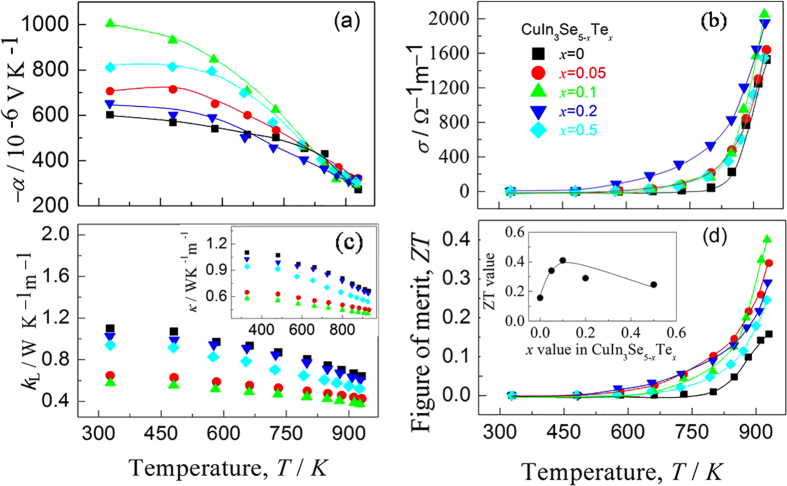
TE properties as function of temperature, (**a**) Seebeck coefficient (*α*); (**b**) Electrical conductivity (*σ*); (**c**) lattice thermal conductivity (*κ*_L_), where an inset is the total *κ*; (**d**) ZT values, an inset is the relation of ZT value with Te content (*x* value).

Since the carrier concentration is inversely related to the Seebeck coefficient (*α*), it is believed that there is a specific Te content at which the maximum *α* value can be attained. Surprisingly, in the present work the sample at *x* = 0.1 has the highest Seebeck coefficients among all samples below ~825 K (Fig. 4a), although the *α* values for different samples converge at high temperatures. The sample at *x* = 0.1 gives the highest *σ* value at ~930 K, at which the *α* and *σ* values are −292.58 *μ*V.K^−1^ and 2.05 × 10^3^ Ω^−1^ m^−1^ respectively (Fig. 4b). The lattice contribution *κ*_L_ as a function of temperature is shown in Fig. 4c, where the *κ*_L_ value reduces with temperature increasing for all the samples. The *κ*_L_ value at *x* = 0.1 is the lowest over the entire temperature range, and at *T* = 930 K it gives 0.37 WK^−1^ m^−1^. At *x* ≥ 0.1 the *κ*_L_ increases with *κ*_L_* = *0.61 WK^−1^ m^−1^ (*x* = 0.2) and 0.52 WK^−1^ m^−1^ (*x* = 0.5). An insert is the total thermal conductivity *κ*, which has a similar composition dependence with *κ*_L_, suggesting the heat carrying by phonons dominates in these compounds.

Combining the three physical parameters (*α, σ* and *κ*), the dimensionless figure of merit (ZT) is shown in Fig. 4d. The highest ZT value of the sample at *x* = 0.1 is 0.4 at 930 K. An insert is the plot of ZT against *x* value in CuIn_3_Se_5−*x*_Te_*x*_. Although this ZT value is still lower than those of other chalcopyrites, such as CuGaTe_2_-based (ZT = 1.22 at 850 K^13^; 0.91~1.07 at 703 K^17^,^19^,^38^; 1.4 at 940 K^39^), AgInSe_2_-based alloys (1.05@815 K^18^, and CuInTe_2_-based (0.69@737 K^40^, 1.18 and 1.3 at 850 K^41^,^42^), it is ~2.6 times that of intrinsic CuIn_3_Se_5_ (ZT = ~0.15).

Upon the substitution of element Te for Se, we engineer the crystal structure (Fig. 1). At *x* = 0.1, there is a relatively big difference between *d*_Cu-Se_ and *d*_In-Se_, along with the highest carrier concentration. Therefore, the lowest *κ*_L_ values are mainly due to the heavy phonon scattering caused by the lattice distortion, coupled with the phonon-carrier interaction. However, the phonon-carrier scattering strength is relatively less important in the temperature range of the present study, therefore, the phonon scattering in the lattice defects plays a major role in reducing the *κ*_L_ value. Furthermore, both the Seebeck coefficient and carrier concentration reach the highest at *x* = 0.1 simultaneously, which is not consistent with the common relationship between the Seebeck coefficient and carrier concentration. We believe that the origin might be due to the alteration of the band structure.

In order to gain a deep understanding of the band structure upon Te incorporation, the Pisarenko plots are shown in Fig. S3, assuming m* = 0.006, 0.08, 0.11 and 0.34*m*_e_ at RT respectively. The data from Jocob^36^ and Dejlle *et al*.^27^ are plotted together for comparison. The *α* values for the Te-incorporated samples at RT slightly increase with *n*_H_ increasing, but the Pisarenko relations can exactly capture the measured values of Seebeck coefficient under assumed effective masses m*. In this sense, the alteration of the bandgap should be taken into consideration.

**Figure 5:**
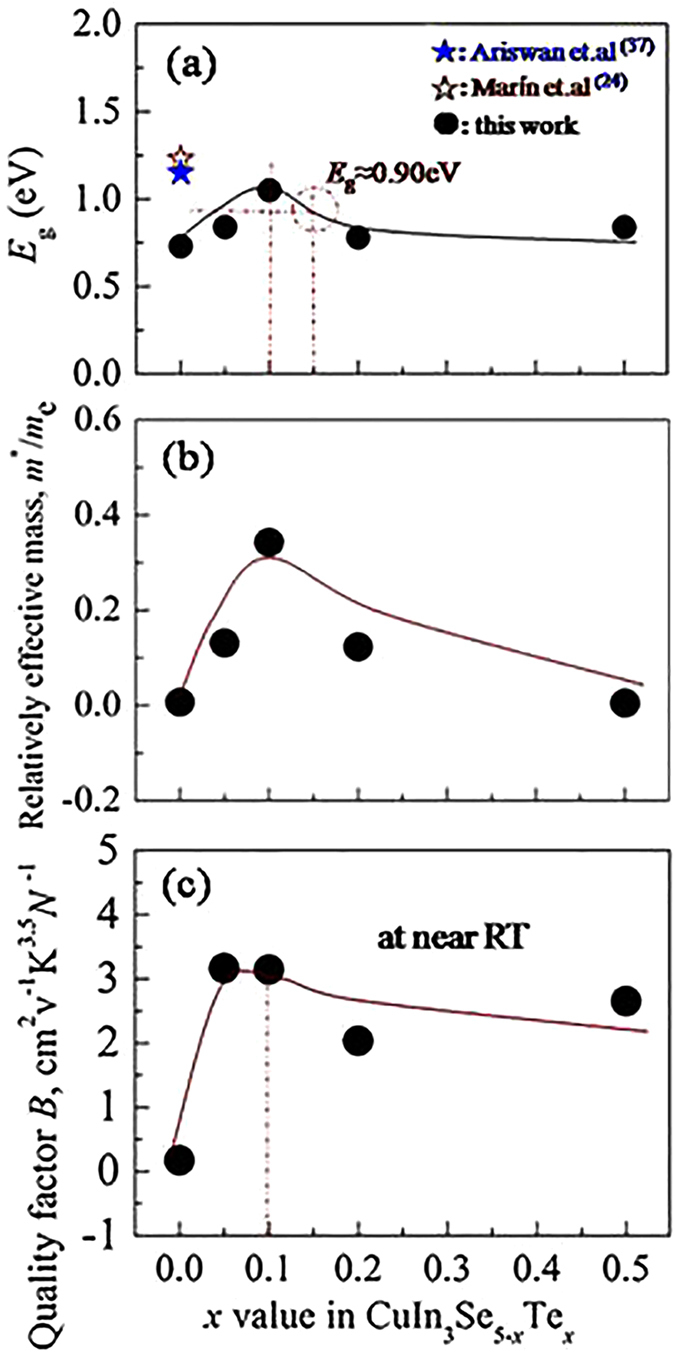
(**a**) *E*_g_ value, roughly estimated using *E*_g_ = 2*α*_max_*eT*, against *x* value, which increases with *x* value increasing until at *x* = 0.1. At *x* ≈ 0.15 *E*_g_ ≈ 0.90 eV. The *E*_g_ values from Ariswan^37^ and Marín *et al*.^24^ are presented for comparison.; (**b**) The relatively effective mass (m*/*m*_e_) as a function of Te content. At *x* = 0.1 the highest m*/*m*_e_ value is obtained; (**c**) The quality factor *B* at RT, defined by *B* = *μ*_H_(m*/*m*_e_)^3/2^*T*^5/2^/*κ*_L_, as a function of *x* value. The *B* value increases until at *x* = 0.1, and then decreases with *x* increasing.

Figure 1c shows the simultaneous elongation of the bond lengths *d*_Cu-Se_ and *d*_In-Se_. This elongation is mainly caused by the increased repulsion between cation and anion upon Te substitution for Se, due to the lower electronegativity of Te (2.1) than that of Se (2.5), thus increasing the covalency degree^43^. In fact, the bond length *d*_Cu-Se_ increases more rapidly than *d*_In-Se_ with Te content increasing. As consequence, when *x* exceeds ~0.15 the bond length *d*_Cu-Se_ is longer than *d*_In-Se_. This suggests that the hybridization (bonding) between Cu-*d* and Se-*p* near the bandgap region has been weakened, resulting in the increased bandgap energy^44^,^45^,^46^. To confirm this result, the bandgap (*E*_g_) is estimated as a function of *x* value using *E*_g_ = 2*α*_max_*eT*^47^, here *T* is the temperature at which *α* appears with the maximum value. Although such an estimation could result in a large deviation of *E*_g_^48^, it can roughly estimate the variation tendency of the *E*_g_ value with chemical composition in some chalcopyrites, for example, the variation tendency of the *E*_g_ value with Mn content in Mn-incorporated Cu_3_Ga_5_Te_9_ system^49^. The results are shown in Fig. 5a. The *E*_g_ values obtained in this work are still less than reported by Ariswan (1.15 eV)^37^ and Marín (1.23 eV)^24^. However, it is observed that the bandgap (*E*_g_) is rapidly enlarged with Te content increasing, and at *x* = 0.1 (∆*u* = 0.15 × 10^−3^) it reaches the highest value of 1.05 eV. Above *x* = 0.1 it is gradually reduced with Te content increasing. At *x* ≈ 0.15 (∆*u* = 0) the *E*_g_ value is estimated to be ~0.90 eV. The reduction in *E*_g_ above *x* ≥ 0.1 might be due to the reduced ionicity upon Te substitution for Se, proposed by Honeyman after he observed that energy gap increases with the crystalline ionicity increasing^50^. Since the high energy gap could prevent the degradation of thermoelectric power through inhibiting the formation of the thermally activated minority carriers^6^,^51^, that is why we have observed the highest Seebeck coefficients (Fig. 4a) and carrier concentration (Fig. 3) at *x* = 0.1. In addition, upon the substitution of Te for Se the relatively effective mass of the carrier (m*/*m*_e_) increases rapidly with Te content from m*/*m*_e_ = 0.007 (*x* = 0) to m*/*m*_e_ = 0.342 (*x* = 0.1) (*m*_e_: mass of the electron), and then decreases with Te content increasing at *x* ≥ 0.1, see Fig. 5b, which suggests that a proper substitution of Te could optimize the band structures.

**Figure 6:**
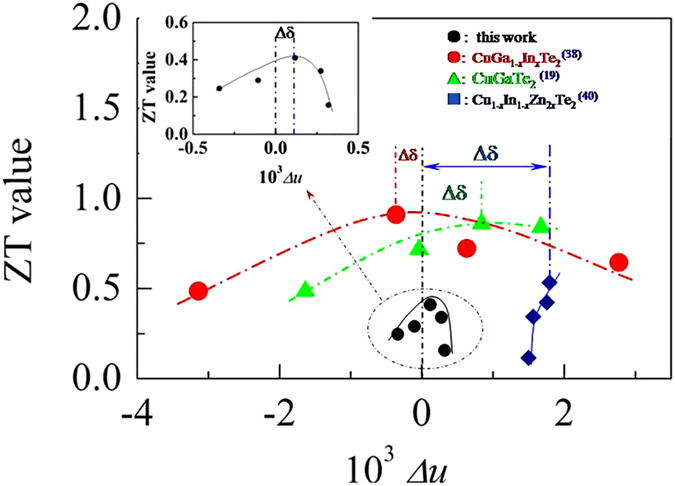
The ZTs against ∆*u*. The maximum ZTs from different chalcopyrites, such as, CuGa_1−*x*_In_*x*_Te_2_^38^, annealed CuGaTe_2_^19^ and Cu_1−*x*_In_1−*x*_Zn_2*x*_Te_2_^40^ are presented for comparison. These chalcopyrite compounds all show that they do not have the highest ZTs at ∆*u* = 0, but at a certain ∆*u* value. An insert is close-up view of present relation between ZTs and ∆*u*. The ZT value reaches the highest at ∆*u* = 0.15 × 10^−3^, rather than at ∆*u* = 0.

Since the chalcopyrite compound at *x* = 0.1 has the highest m*/*m*_e_ value, carrier concentration and lowest *κ*_L_ value, hence a highest quality factor *B (B* = *μ*_H_(m*/*m*_e_)^3/2^*T*^5/2^/*κ*_L_)^12^ can be anticipated. We therefore plot *B* value at near RT as a function of *x*, shown in Fig. 5c. In Fig. 5c we observed that the *B* value rapidly increases with *x* value increasing until *x* = 0.1, and then it decreases. Therefore, we consider that it is not necessary to let *x* > 0.1 (∆*u* = 0.15 × 10^−3^ at *x* = 0.1) in order to improve the TE performance. When *x* > 0.1 (for example: ∆*u* → 0 when *x* → 0.15), the TE performance may degrade owing to the enhanced lattice contribution *κ*_L_. In fact, the ZT value reaches the highest at *x* = 0.1 (see the insert in Fig. 4d), corresponding to ∆*u* = 0.15 × 10^−3^. ZT value vs ∆*u* is plotted in Fig. 6, where the ZTs against ∆*u* in other chalcopyrite compounds, such as, CuGa_1−*x*_In_*x*_Te_2_^38^, annealed CuGaTe_2_^19^ and CuIn_1−*x*_Zn_*x*_Te_2_^40^, are also presented for comparison. It is worth noting that at ∆*u* = 0, corresponding to *x* = 0.15, neither the *E*_g_ (*E*_g_ ≈ 0.90 eV) nor the ZT value reaches the highest, see Figs 5a and 6. The insert in Fig. 6 is close-up view of present relation between ZTs and ∆*u* in this work. These chalcopyrite compounds all show that they do not have the highest ZTs at ∆*u* = 0, but at a certain ∆*u* value, i.e. at a distance (∆δ) from ∆*u* = 0 they give the highest ZTs. This indicates that the best TE performance can be achieved with a certain crystal structure parameters for each chalcopyrite compound with a specific space group. This finding is in good agreement with the previous assumption that when ∆*u* = 0 or *η* = 1 it is difficulty to achieve the highest TE performance for some chalcopyrite compounds, due not only to electrical inactivity, but also a relatively high lattice part *κ*_L_.

## Conclusions

In this work the chalcopyrite compounds CuIn_3_Se_5−*x*_Te_*x*_ (*x* = 0~0.5) with space group 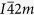 have been prepared, and the dilation of the crystal structure as *x* value increases has been observed. At *x* = 0.15 the anion position displacement ∆*u* is tending to be zero. But at *x* = 0.1 (∆*u* = 0.15 × 10^−3^) both the band energy (*E*_g_) and the Hall carrier concentration *n*_H_ reach the highest values, leading to the best thermoelectric performance with the ZT value of 0.4 at 930 K. Furthermore, at *x* = 0.1 it is the critical Te content in CuIn_3_Se_5−*x*_Te_*x*_ (*x* = 0~0.5), above which the quality factor *B* decreases, and lattice thermal conductivity *κ*_L_ are higher than those at *x* = 0.1, caused by reduced phonon scattering in lattice defects. We therefore conclude that the best thermoelectric performance can be achieved at a certain ∆*u* value (∆*u* ≠ 0) if one can engineer the crystal structures of the chalcopyrite with a specific space group.

## Additional Information

**How to cite this article**: Lu, Y. *et al*. Enhanced thermoelectric performance of a chalcopyrite compound CuIn_3_Se_5−*x*_Te_*x*_ (*x* = 0~0.5) through crystal structure engineering. *Sci. Rep.* **7**, 40224; doi: 10.1038/srep40224 (2017).

**Publisher's note:** Springer Nature remains neutral with regard to jurisdictional claims in published maps and institutional affiliations.

## Supplementary Material

Supplementary Figures

## References

[b1] Hochbaum Allon, I. . Enhanced thermoelectric performance of rough silicon nanowires. Nature 451, 163–167 (2008). 18185582 10.1038/nature06381

[b2] Boukai A. I. . Silicon nanowires as efficient thermoelectric materials. Nature 451, 168–171 (2008). 18185583 10.1038/nature06458

[b3] Poudel B. . High-thermoelectric performance of nanostructured bismuth antimony telluride bulk alloys. Science 320, 634–638 (2008). 18356488 10.1126/science.1156446

[b4] Pei Y. . Convergence of electronic bands for high performance bulk thermoelectrics. Nature 473, 66–69 (2011). 21544143 10.1038/nature09996

[b5] Heremans J. P. . Enhancement of thermoelectric efficiency in PbTe by distortion of the electronic density of states. Science 321, 554–557 (2008). 18653890 10.1126/science.1159725

[b6] Pei Y. . Band engineering of thermoelectric materials. Adv. Mater. 24, 6125–6135 (2012). 23074043 10.1002/adma.201202919

[b7] Zhao L. . Ultrahigh power factor and thermoelectric performance in hole-doped single-crystal SnSe. Science 351, 141–144 (2016). 26612831 10.1126/science.aad3749

[b8] Zhao L. . Ultralow thermal conductivity and high thermoelectric figure of merit in SnSe crystals. Nature 508, 373–389 (2014). 24740068 10.1038/nature13184

[b9] Liu W. . Convergence of conduction bands as a means of enhancing thermoelectric Performance of n-Type Mg_2_Si_1−*x*_Sn_*x*_solid solutions. Phys. Rev. Lett. 108, 166601 (2012). 22680741 10.1103/PhysRevLett.108.166601

[b10] Liu X. . Low Electron scattering potentials in high performance Mg_2_Si_0.45_Sn_0.55_ based thermoelectric solid solutions with band convergence. Adv. Energy Mater. 3, 1238–1244 (2013).

[b11] Yu C. . High-performance half-Heusler thermoelectric materials Hf_1−*x*_Zr_*x*_NiSn_1−*y*_Sb_*y*_ prepared by levitation melting and spark plasma sintering. Acta Mater. 57, 2757–2764 (2009).

[b12] Shi X. . Recent advances in high-performance bulk thermoelectric materials. Inter. Mater. Rev. 61, 379–415 (2016).

[b13] Zhang J. . High-performance pseudocubic thermoelectric materials from non-cubic chalcopyrite compounds. Adv. Mater. 26, 3848–3853 (2014). 24692165 10.1002/adma.201400058

[b14] Zhang S. B. . Stabilization of ternary compounds via ordered arrays of defect pairs. Phys. Rev. Lett. 78, 4059–4062 (1997).

[b15] Rincόn C. . Scattering of the charge carriers by ordered arrays of defect pairs in ternary chalcopyrite semiconductors. Appl. Phys. Lett. 80, 998–1000 (2002).

[b16] Wei S. H. . Effects of Ga addition to CuInSe_2_ on its electronic, structural, and defect properties. Appl. Phys. Lett. 72, 3199–3201 (1998).

[b17] Cui J. . Promising defect thermoelectric semiconductors of Cu_1−*x*_GaSb_*x*_Te_2_ (*x* = 0–0.1) with the chalcopyrite structure. J. Mater. Chem. A 1, 677–683 (2013).

[b18] Wang L. . Site Occupations of Zn in AgInSe_2_-based chalcopyrites responsible for modified structures and significantly improved thermoelectric performance. RSC Adv. 4, 33897–33904 (2014).

[b19] Wu W. C. . Manipulation of the crystal structure defects: An alternative route to the reduction In lattice thermal conductivity and improvement in thermoelectric performance of CuGaTe_2_. Appl. Phys. Lett. 103, 011905 (2013).

[b20] Hanada T. . Crystal structure of CuIn_3_Se_5_ semiconductor studied using electron and X-ray diffractions. Jpn, J. Appl. Phys. 36, L1494–L1497 (1997).

[b21] Xiao H. Z. . Structural, optical, and electrical properties of epitaxial chalcopyrite CuIn_3_Se_5_ films. J. Appl. Phys. 76, 1503–1510 (1994).

[b22] Hernández E. . Electrical properties of CuIn_3_Se_5_ bulk crystal at low temperature. Cryst. Res. Technol. 37, 1088–1093 (2002).

[b23] Chang C. *et al. et al*. Local structure of CuIn_3_Se_5_: X-ray absorption fine structure study and first-principles calculations. Phys. Rev. B 68, 054108 (2003).

[b24] Marín G. . X-ray powder diffraction and optical characterization of the Cu(In_1−*x*_Ga_*x*_)_3_Se_5_ semiconducting system. Mater. Res. Bull. 33, 1057–1068 (1998).

[b25] Philip R. R. & Pradeep B. Nonideal anion displacement, band gap variation, and valence band splitting in Cu-In-Se compounds. Thin Solid Films 472, 136–143 (2005).

[b26] Díaz R. & Arranz A. Effect of the composition on the ionic motion in an In-rich chalcopyrite ingot of the Cu-Ag-In-Se system. J. Alloys Compds. 590, 80–86 (2014).

[b27] Djellal L. . Physical, photoelectrochemical properties of CuIn_3_Se_5_ and relevance for hydrogen production. Mater. Chem. Phys. 137, 340–345 (2012).

[b28] Paszkowicz W. . Rietveld refinement for CuInSe_2_ and CuIn_3_Se_5_. J. Alloys Compds. 362, 241–247 (2004).

[b29] Abrahams S. C. & Bernstein J. L. Piezoelectric Nonlinear Optic CuGaS_2_ and CuInS_2_ Crystal Structure: Sublattice Distortion in A^I^B^III^C_2_^VI^ and A^II^B^IV^C_2_^V^ Type Chalcopyrites. J. Chem. Phys. 59, 5415–5422 (1973).

[b30] Abrahams S. C. & Bernstein J. L. Piezoelectric Nonlinear Optic CuGaSe_2_ and CdGeAs_2_: Crystal Structure, Chalcopyrite Microhardness, and Sublattice Distortion. J. Chem. Phys. 61, 1140–1146 (1974).

[b31] Jaffe J. E. & Zunger A. Electronic structure of the ternary chalcopyrite semiconductors CuAlS_2_, CuGaS_2_, CuInS_2_, CuAlSe_2_, CuGaSe_2_, and CuInSe_2_. Phys. Rev. B 28, 5822–5847 (1983).

[b32] Yao J. . Site preference of manganese on the copper site in Mn-Substituted CuInSe_2_ chalcopyrites Revealed by a combined neutron and x-ray powder diffraction study. Chem. Mater. 22, 1647–1655 (2010).

[b33] Boehnke U. C. & Kühn G. Phase relations in the ternary system Cu-ln-Se. J. Mater. Sci. 22, 1635–1641 (1987).

[b34] Yao J. . Effects of Mn substitution on the structure and properties of chalcopyrite-type CuInSe_2_. J. Solid State Chem. 182, 2579–2586 (2009).

[b35] Wang H. P. . Studies on monocrystalline CuInSe_2_ and CuIn_3_Se_5_. Thin Solid Films 361–362, 494–497 (2000).

[b36] Jacob R. . Optoelectronic and low temperature thermoelectric effects in the OVC n-CuIn_3_Se_5_ thin films. Phys. Sta. Sol. A 209, 2195–2200 (2012).

[b37] Ariswan . Structural, optical and electrical properties of the ordered vacancy compound CuIn_3_Se_5_ thin films fabricated by flash evaporation. Solid State Commun. 124, 391–396 (2002).

[b38] Li Y. . High thermoelectric performance of solid solutions CuGa_1−*x*_In_*x*_Te_2_ (*x* = 0–1.0). Appl. Phys. Lett. 100, 231903 (2012).

[b39] Plirdpring T. . Chalcopyrite CuGaTe_2_: A high-efficiency bulk thermoelectric material. Adv. Mater. 24, 3622–3626 (2012). 22689017 10.1002/adma.201200732

[b40] Yang J. . Lattice defects and thermoelectric properties: the case of p-type CuInTe_2_ chalcopyrite on Introduction of Zinc. Dalton Trans. 43, 15228–15235 (2014). 25187213 10.1039/c4dt01909a

[b41] Liu R. . Ternary compound CuInTe_2_: a promising thermoelectric material with diamond-like structure. Chem. Commun. 48, 3818–3820 (2012). 10.1039/c2cc30318c 22414929

[b42] Carr Winston D. & Morelli Donald T. Influence of doping and solid solution formation on the thermoelectric properties of chalcopyrite semiconductors. J. Alloys Compds. 630, 277–281 (2015).

[b43] Ma C. G. & Brik M. G. First principles studies of the structural, electronic and optical properties of LiInSe_2_ and LiInTe_2_ chalcopyrite crystals. Solid State Commun. 203, 69–74 (2015).

[b44] Ozaki S. & Adachi S. Optical absorption and photoluminescence in the ternary chalcopyrite semiconductor AgInSe_2_. J. Appl. Phys. 100, 113526 (2006).

[b45] Shay J. L. . Electronic Structure of AlInSe_2_ and CuInSe_2_. Phys. Rev. B 7, 4485–4490 (1973).

[b46] Shay J. L. & Kasper H. M. Direct Observation of Cu *d* Levels in I-III-VI_2_ Compounds. Phys. Rev. Lett. 29, 1162–1164 (1972).

[b47] Goldsmid H. J. & Sharp J. W. Estimation of the thermal band gap of a semiconductor from Seebeck measurements. J. Elect. Mater. 28, 869–872 (1999).

[b48] Gibbs Z. M. . Band gap estimation from temperature dependent Seebeck measurement—deviations from the 2e|S|_max_T_max_ relation. Appl. Phys. Lett. 106, 022112 (2015).

[b49] Cui J. . Engineering the energy gap near the valence band edge in Mn-incorporated Cu_3_Ga_5_Te_9_ for an enhanced thermoelectric performance. J. Mater. Chem. C, 4, 8014 (2016).

[b50] Honeyman W. N. & Wilkinson K. H. Growth and properties of single crystals of group I–III–VI_2_ ternary semiconductors. J. Phys. D: Appl. Phys. 4, 1182–1185 (1971).

[b51] Pei Y. . Stabilizing the optimal carrier concentration for high thermoelectric efficiency. Adv. Mater. 23, 5674–5678 (2011). 22052689 10.1002/adma.201103153

