# Supplementary material for: Enhanced thermoelectric performance of a chalcopyrite compound CuIn3Se5−xTex (x = 0~0.5) through crystal structure engineering
Source: Sci Rep. 2017 Jan 6;7:40224. doi: 10.1038/srep40224 (PMC5216356; doi:10.1038/srep40224)
Supplement: Supplementary Figures [file srep40224-s1.pdf]

Supporting information

Enhanced thermoelectric performance of a chalcopyrite compound $CuIn_3Se_{5-x}Te_x$ (x=0~0.5) through crystal structure engineering

Yufu Lu[a,b], Shaoping Chen[a,*], Wenchang Wu,[b] Zhengliang Du[b], Yimin Chao[c,*], Jiaolin Cui[b,*]

[a] School of Materials & Chemical Engineering, Ningbo University of Technology, Ningbo 315016, China

[b] Yufu Lu, Shaoping Chen: Materials Science and Engineering College, Taiyuan University of Technology, Taiyuan, 030024, China.

[c] School of Chemistry, University of East Anglia, Norwich, NR4 7TJ, United Kingdom

Corresponding author's contact information:

Jiaolin Cui:

315016, School of Materials, Ningbo University of Technology, Ningbo 315016, China

E-mail: cuijiaolin@163.com

Tel: 86-574-87080504

Shaoping Chen

Materials Science and Engineering College, Taiyuan University of Technology, Taiyuan, 030024, China.

E-mail: sxchenshaoping@163.com

Yimin Chao

School of Chemistry, University of East Anglia, Norwich NR4 7TJ, United Kingdom

E-mail:Y.Chao@uea.ac.uk

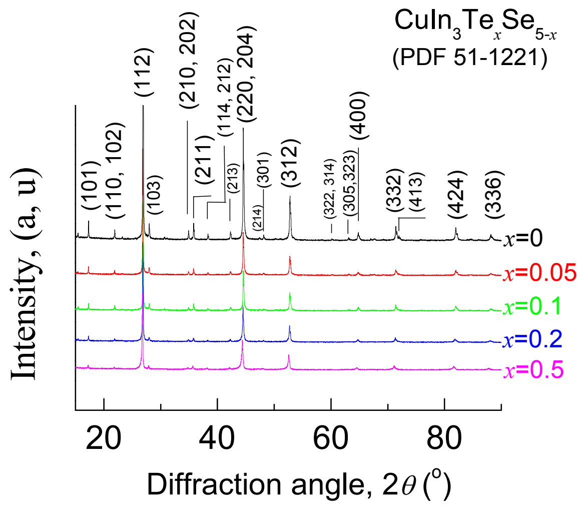

Fig.S1 X-ray diffraction patterns of $CuIn_3Se_{5-x}Te_x$ powders.

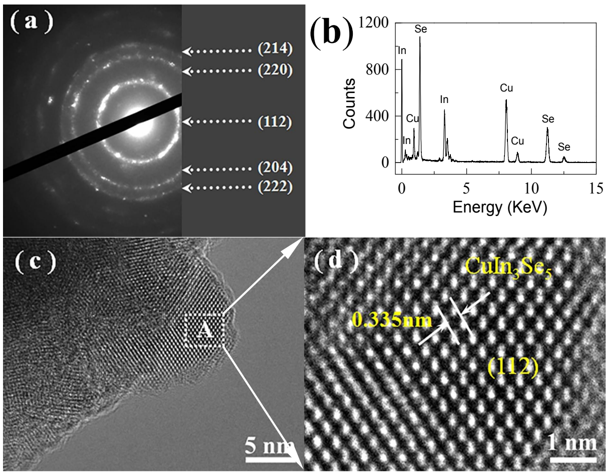

Fig.S2 High resolution transmission electron microscopy (HRTEM) image observed in $CuIn_3Se_5$; (a) The selected area electron diffraction (SAED) pattern; (b) Chemical compositions analyses; (c) High resolution TEM image; (d) Magnified high resolution TEM image, which shows that the spacing between (112) crystal planes is about 0.335nm.

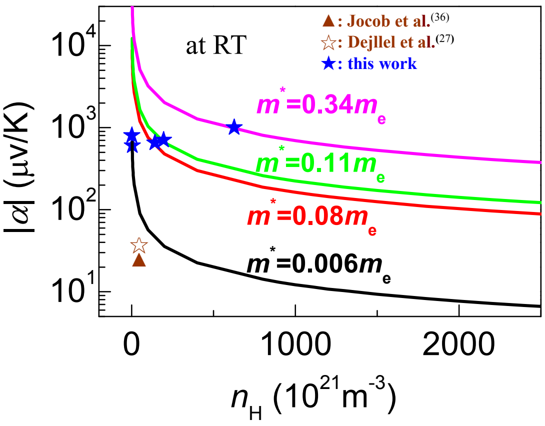

Fig.S3 Pisarenko plots assuming m^* =0.006, 0.08, 0.11 and 0.34m_e at RT respectively. The results from Jocob[36] and Dejllei[27] et al. are presented for comparison.
